# Canopy and Ear Traits Associated With Avoidance of Fusarium Head Blight in Wheat

**DOI:** 10.3389/fpls.2018.01021

**Published:** 2018-07-31

**Authors:** Stephen Jones, Arifa Farooqi, John Foulkes, Debbie L. Sparkes, Robert Linforth, Rumiana V. Ray

**Affiliations:** ^1^Division of Plant and Crop Sciences, School of Biosciences, University of Nottingham, Nottingham, United Kingdom; ^2^Division of Food Sciences, School of Biosciences, University of Nottingham, Nottingham, United Kingdom

**Keywords:** Fusarium head blight, disease avoidance, wheat, ear and canopy traits, pathogen DNA, mycotoxins

## Abstract

Doubled haploid and elite wheat genotypes were ground inoculated in three field experiments and head spray inoculated in two glasshouse experiments, using mixed *Fusarium* and *Microdochium* species, to identify crop canopy and ear traits associated with Fusarium head blight (FHB) disease. In all experiments, flag leaf length and tiller number were consistently identified as the most significant canopy traits contributing to progression of FHB caused by *Fusarium graminearum, F. culmorum*, and *F. avenaceum*. The influence of ear traits was greater for *F. poae* that may possess more diverse routes for transmission and spread. Consistently, spikelet density was associated with increased disease severity in the field. *F. graminearum, F. culmorum*, and *F. langsethiae* were the main mycotoxin producers and their respective toxins were significantly related to fungal biomass and number of spikelets per ear. Genotypes with lower tiller numbers, shorter flag leaves and less dense ears may be able to avoid FHB disease caused by *F. graminearum, F. culmorum, F. avenaceum*, or *Microdochium* species however selection for these canopy and ear architectural traits to enable disease avoidance in wheat is likely to result in a potential trade-off with grain yield and therefore only moderately advantageous in susceptible genotypes.

## Introduction

Fusarium head blight (FHB) is a devastating fungal disease of wheat and other small grain cereals worldwide caused by a complex of toxigenic *Fusarium* spp. (Logrieco et al., 2002) and non-toxigenic *Microdochium* spp. (Glynn and Edwards, 2010). Infection occurs during anthesis (GS 61-69) (Zadoks et al., 1974) and leads to a reduction of yield, grain quality, and the production of harmful mycotoxins to humans and animals by *Fusarium* spp. (Marin et al., 2013). Control methods for FHB include crop rotation, deep cultivation, and fungicide application. However, the development of cultivars with improved FHB resistance is considered the most sustainable method for controlling the impact of this disease (Edwards, 2004).

Resistance to FHB is a quantitative trait conferred by multiple genes with minor to moderate effects (Buerstmayr et al., 2009), however less is known of the influence of plant morphological traits to passive disease resistance or susceptibility. Passive disease resistance acts independently of the physiological status of the host relying on intrinsic traits that may allow the plant to avoid or escape disease. Whilst avoidance is a defense mechanism under genetic control, disease escape is mostly associated with morphological traits that may result in reduced likelihood of contact between the pathogen and usually a susceptible host and by definition does not rely on the function of disease resistance genes.

Plant height was first identified as a potential FHB disease escape trait with taller plants having significantly less severe symptoms possibly due to the reduced dispersal of ground inoculum to the ear (Mesterhazy, 1995; Hilton et al., 1999). Numerous studies thereafter (Paillard et al., 2004; Steiner et al., 2004; Schmolke et al., 2008; Szabo-Hever et al., 2014) identified quantitative trait loci (QTL) associated with FHB resistance and plant height, and Srinivasachary et al. (2008) showed that increased FHB susceptibility in shorter genotypes was genetically linked with *Rht1* dwarfing alleles. Significant correlations between ear traits such as length (Suzuki et al., 2012) or density (Steiner et al., 2004) and FHB susceptibility rather than resistance have been most often reported (Somers et al., 2003; Schmolke et al., 2005; Liu et al., 2007). Studies investigating the influence of awns on FHB also reported conflicting results, of an increase (Mesterhazy, 1995; Liu et al., 2013), a decrease (Buerstmayr et al., 2000; Giancaspro et al., 2016) in FHB, or no relationship at all (Buerstmayr et al., 2002; Liu et al., 2007) despite overlapping quantitative trait loci on the long arm of chromosome 5A associated with FHB resistance and the gene *B1*, controlling the presence of awns (Gervais et al., 2003; Liu et al., 2013). The most consistent ear trait being extensively shown to have a negative effect on the extent of FHB infection has been the degree of anther extrusion (Buerstmayr et al., 2012; Lu et al., 2013; He et al., 2014). Whilst most previous studies focussed on ear traits, information on the role of canopy traits potentially enabling disease escape or resulting in increased susceptibility to FHB is lacking.

In the field, where diverse FHB pathogen populations exist (Nielsen et al., 2014) morphological traits on an individual or population level are likely to contribute significantly to disease outcome by impacting on the pathogen population structure, co-existence and predominance. Canopy and ear traits therefore interacting with the environment are likely to ultimately influence mycotoxin accumulation in grain by affecting the ability of the producers to compete in reaching the ear thus resulting in niche exclusion for the deterred species. Understanding the ideotype of plant traits leading to disease susceptibility or escape through environmental interactions can improve agronomic measures for disease control.

The present study aimed to identify for the first time canopy and ear traits in wheat associated with disease avoidance or susceptibility to FHB, caused by a diverse population of *Fusarium* and *Microdochium* spp. First, ground inoculated, field experiments using limited number of phenotypically divergent genotypes were used to enable canopy and ear passive disease traits to be expressed and identified under natural rainfall conditions. Secondly, the influence of canopy traits to disease was negated using spray-head inoculations in glasshouse experiments to determine if any of the identified traits were associated with genetic resistance. Lastly, a larger population of genotypes from the same population cross was used to confirm and test the identified traits under artificial misting creating severe disease pressure. Predictions of FHB disease were made using pathogen DNA and mycotoxin accumulation determined by quantitative Real-time PCR and LCMS/MS, respectively, while canopy and ear traits were identified using principal component analysis.

## Materials and methods

### Plant material

The doubled haploid (DH) population created with the maize pollination technique (Laurie and Bennett, 1986) used in this study originated from a cross between an adapted UK hard winter wheat Rialto [pedigree: Haven/Fresco (489936)], released in 1995 by RAGT Seeds Ltd., and spring wheat advanced breeding line L8 (CMH8OA.763-1B-1Y-2B-3Y-OY) that is a “large spike” phenotype derived in a wide-crossing programme by the International Maize and Wheat Improvement Centre (CIMMYT) involving *Agropyron elongatum, Triticum polonicum*, and *Triticum aestivum* (var. Morocco) to create restructured hexaploid wheat plant types exploiting heterosis (Rajaram, 2001). A selection of 10 lines from this population was chosen for use within the first two experiments in 2010 and 2011 based upon their wide expression of traits such as plant height, canopy density, ear length, and spikelet number, using a previous field data set (Foulkes. pers. comm.). The UK winter wheat elite varieties Ambrosia, Claire, Grafton, Rialto and Solstice were also included in each experiment to make a total of 15 genotypes. The elite varieties included in the experiments were selected to vary in their resistance scores for FHB based on the recommended list of UK varieties (AHDB) whilst DH lines were selected to vary in canopy and ear traits. The field experiment in 2012 utilized a set of the DH population of 86 lines, including the parents.

### Inoculum production

Ground inoculum production mimicking as close as possible diverse pathogen FHB complexes utilized isolates of *F. graminearum* (212, 241, 214), *F. culmorum* (218, 215, 236), *M. majus* (213, 211, 224), *M. nivale* (226, 222), *F. poae* (246, 245, 232), *F. avenaceum* (248, 210, 235), and *F. langsethiae* (243, 247, 221) in 2010 and 2011 whereas in 2012 the latter three species were excluded. Isolates were sub-cultured onto potato dextrose agar plates and, after 7 days, five plugs were removed from each actively growing culture to inoculate a conical flask containing 100 mL of sterile potato dextrose broth (PDB) (Sigma-Aldrich, UK) (Tuite, 1969). After 1 week, agar discs (5–7 mm in diameter) cut from the margins of each actively growing isolates colonies were placed into a conical flask containing 120 mL sterile potato dextrose broth (PDB, Sigma-Aldrich, UK). Each flask was sealed with sterile cotton wool and foil, placed on an orbital mixer and left in darkness for 5 days at 20°C at 90 RPM. Six hundred and fifty grams of oats and 65 ml of deionized water were placed into 40 separate bags (one bag for each isolate) and autoclaved at 123°C for 1 h, cooled overnight and the autoclave process repeated the following day. Once ready, 50 mL of the inoculated PDB was added to each bag of oats, thoroughly mixed, and incubated for 14 days to create a composite inoculum.

### Field experiments and ground inoculation

The present study used three field experiments performed in 2009–10, 2010–11, and 2011–12 using elite lines and genotypes from the DH population and the parental lines (Data Sheet 1). Field experiments were undertaken at experiment sites located at the University of Nottingham, Sutton Bonington Campus, UK (latitude 52.83368, longitude −1.24638). Drilling of the field experiments was carried out on the 15 October 2010, 11 October 2011, and 9 October 2012, respectively, using a seed rate of 375 seeds/m^2^.

The experimental design of 2010 and 2011 experiments was a randomized block design in 6.0 × 2 m plot in three replicates. In 2012 due to the greater number of genotypes, plot size was reduced to 1 m^2^ (1 × 1 m) plot and again the experiment used a randomized block design with three replicates. Fungicide strategy was aimed at robust control of foliar pathogens, whilst minimizing negative effects on *Fusarium* or *Microdochium* species, except at GS30 (Zadoks et al., 1974) where fungicide was used to remove any background *Fusarium* or *Microdochium* inoculum prior to inoculation at GS31. At GS30 (Zadoks et al., 1974), the experiments were treated with 0.75 l/ha of Amistar Opti® (100 g/l azoxystrobin + 500 g/l chlorothalonil) and 0.75 l/ha Folicur®(250 g/l tebuconazole) in 2010 and 0.5 l/ha Flexity® (300 g/l metrafenone), 1 l/ha Amistar® (250 g/l azoxystrobin) and 0.75 l/ha of Alto Elite® (40 g/l cyproconazole + 375 g/l chlorothalonil) in 2011 and 2012. At GS31, treatment was made against eyespot or foliar pathogens using 1 l/ha of Tracker® (233 g/l boscalid + 67 g/l epoxiconazole) and 0.5 l/ha of Flexity® in 2010 and 0.5 l/ha Comet® 200 (200 g/l pyraclostrobin), 0.15 l/ha Vegas® (50 g/l cyflufenamid) and 1 l/ha of Bravo® 500 (500 g/l of chlorothalonil) in 2011 and 2012. At GS39, 1 l/ha Corbel® (750 g/l fenpropimorph) and 2 l/ha Alto Elite® (40 g/l cyproconazole + 375 g/l chlorothalonil) were used against foliar diseases.

Each plot of the 2010, 2011, and 2012 field experiments was inoculated manually by ground application of uniformly mixed oat grains colonized by *Fusarium* and *Microdochium* species. The infected oat grains of equal quantities from each species were distributed in the field at GS 31 at a rate of 37.5 g m^−2^. The field experiment in 2012 was mist irrigated every morning at GS65 for 10 min every hour from 7 a.m. to 11 a.m. to stimulate infection. However, artificial misting was not used in the 2010 and 2011 field experiments, allowing FHB epidemics to develop under natural rainfall patterns.

### Evaluation of canopy and ear traits

To evaluate important canopy and ear traits, a 0.25 m^2^ quadrat (0.5 × 0.5 m) of plants was sampled by digging plants up from each plot at GS65 (Zadoks et al., 1974), avoiding the outer rows of the plot and ensuring that each genotype was sampled at the same growth stage. The above-ground plant biomass underwent a detailed growth analysis to determine crop canopy architecture traits. Five plants were selected at random from each sample for the assessments of tiller number, plant height, flag leaf traits, and ear traits. Tiller number was recorded as the total number of fertile shoots (those with an ear) per plant minus the main shoot. Plant height was measured as the distance in centimeters from the base of the stem to the tip of the ear. Flag leaf length (FLL) was measured from the ligule to the leaf tip and flag leaf width (FLW) was measured across the leaf at the midpoint in centimeters. Ear traits included the ear length, measured from the ear collar to the tip, spikelet number was counted as the total number of fertile spikelets per ear and spikelet density calculated as the spikelet number divided by the ear length on all fertile shoots from selected plants. Awn length was measured in centimeters, in the glasshouse experiments only on two randomly selected spikelets on opposing sides of the ear to calculate the mean.

### Glasshouse experiments and spray inoculation

Two glasshouse experiments were undertaken in 2011 and 2012 at The University of Nottingham, Sutton Bonington Campus. Each experiment consisted of four randomized blocks using the same 15 DH and elite wheat lines as in the field in 2010 and 2011 experiments but Solstice and Grafton were replaced with two well-characterized resistance controls, Sumai-3 and Frontana, as one of the main objectives here was to negate the influence of canopy traits and test the resistance responses of the DH lines under controlled head spray inoculation. Seeds were sown into trays and kept in a growth chamber at 8°C for 6 weeks to complete vernalization. One plant of each variety was then transplanted into a 3 L pot filled with John Innes No.3 compost, before being randomized into their individual blocks within the glasshouse. The glasshouse temperature was maintained between 5 and 12°C via a combination of automatic vents and heating system and an automatic watering system was used to water pots twice a day. Powdery mildew was controlled via the use of a sulfur burner at weekly intervals until GS51. Physiological traits were measured *in-situ* at GS65 prior to the spray application of mixed inoculum of *Fusarium* and *Microdochium* isolates to wheat heads.

Spray inoculum production utilized the same isolates of *Fusarium* and *Microdochium* as the ground inoculum for 2010 and 2011. Each isolate was grown on 5 plates of Tap Water Agar under a 12 h regime of near-UV light and darkness for 10 days before harvesting spores. Spore solutions were quantified using a haemocytometer and were then diluted in sterile water to create mixed inoculum solutions of equal quantities of each species, totaling 100,000 spores/ml. Each genotype was monitored daily for GS65, at which point the physiological traits were measured *in-situ*, followed by spray inoculation to the head on the main stem until run off. Inoculated ears were covered with a plastic bag for 24 h to stimulate infection.

### Visual FHB assessment

Within the field experiments, visual disease assessments were carried out on each genotype from mid-anthesis (GS65) onwards at 4 day intervals over a 33 day period until maturity within the ear prevented further assessments. Twenty ears per plot were randomly assessed in which the number of visible symptoms (either dark lesions or bleached spikelets) observed on ears was recorded and divided by the total number of spikelets per ear to measure the initial infection and disease spread over time. Glasshouse visual disease assessments were carried out from mid-anthesis (GS65) onwards at 4 day intervals over a 22 day period using the same methodology as the field visual disease assessment following the same heads over time. From this data the area under the disease progress curve (AUDPC) was also calculated using a mathematical formula originally devised by Shaner and Finney (1977), as shown in Equation (1), where *yi* is the score of visually infected spikelets on the ith day and ti is the day of the ith observation and N is the total number of observations.

(1) $$\begin{array}{r} \text{AUDPC}=\sum_{i\;=\;1}^{Ni-1}\left(\frac{{\text{y}}_{\text{i}}+\;y_{i+1}}{2}\right)\left({\text{t}}_{\text{i}+1}-\;{\text{t}}_{\text{i}}\right) \end{array}$$

### Pathogen DNA extraction and quantification by real-time PCR for *Fusarium* and *Microdochium* spp.

To prevent further increase of *Fusarium* infection and mycotoxin accumulation once physiological maturity had been reached, genotypes were harvested individually by hand once grain ripened (GS92). Each genotype was threshed using a stationary Haldrup LT-20 thresher (Haldrup, Ilshofen, Germany) and the grain milled to fine flour using a Krups F203 grinder (Krups, Windsor). In 2012, replicates of 30 selected lines ranging in AUDPC from 24 to 1,404 were processed for DNA extraction and then 10 g of flour from three replicates of these 30 selected lines was combined and thoroughly mixed together for mycotoxin extraction. Milled grain samples were stored at −20°C until DNA extraction. The DNA extraction method used prior to quantitative real-time PCR was as described by Nielsen et al. (2014).

Harvested grain DNA samples from the field and glass house experiments were analyzed with real time PCR using thermal cycler CFX96 (Bio-Rad, UK) to quantify each pathogen species. DNA standard curves (10^−0^–10^−6^ ng μl^−1^) were created from pure DNA of *F. graminearum* (isolate 212, University of Nottingham), *F. culmorum* (isolate 236, University of Nottingham), *F. avenaceum* (isolate 40, University of Nottingham), *F. poae* (isolate 246, University of Nottingham), *F. langsethiae* (isolate 227, University of Nottingham), *M. majus* (isolate 224, University of Nottingham), and *M. nivale* (isolate 226, University of Nottingham). The amplification mix for each species consisted of 2x iQ SYBR Green Supermix (Bio-Rad, UK) used as per the manufacturer's instructions, plus 250 nM of both forward and reverse primers. Species specific primers used for quantification of fungal DNA, PCR programme and conditions were the same as shown in Nielsen et al. (2014). A template of 2.5 μl DNA was used in a total reaction volume of 12.5 μl. The negative control used 2.5 μl of PCR-grade water in place of the of the DNA template. The limit of detection for all seven assays was 10^−4^ pg ng^−1^ total fungal DNA and all assays had an efficiency of between 95 and 100% (Nielsen et al., 2014).

### Mycotoxin extraction from grain from field experiments and quantification by LCMS/MS

All flour samples from 2010 and 2011 and 30 selected lines from 2012 field experiments were used for mycotoxin extraction and analysis by LCMS/MS as described by Drakulic et al. (2016). Briefly, flour samples (12.5 g) were spiked with ^13^C-labeled deoxynivalenol (DON) internal standard to produce a final concentration of 20 μg kg^−1^ and blended with 50 mL methanol (100%) for 3 min. Samples were filtered through filter paper (Whatman No. 1) and 2 mL of the filtrate made up to 50 mL with PBS buffer and filtered through fiberglass filter. The filtrate (20 mL) was passed over a clean-up column by gravity over a 30 min period, and then the column was washed with sterile water prior to elution of samples by passing through 1 mL methanol (100%). Samples were dried under a stream of nitrogen gas then re-dissolved in 500 μL methanol (10%) in preparation for LCMS/MS.

The LCMS/MS analysis was performed on an Agilent 1100 series LC system coupled to a triple quadrupole Micromass Quattro Ultima V4.0 SP4 (Waters) and equipped with Luna® C18 Å100 LC (5 μm, 250 × 3 mm) column. The flow rate was 0.5 mL min^−1^ and the injection volume was 30 μL. Mobile phase A was 90% water with 10% methanol, and mobile phase B was 100% methanol. A linear binary gradient was applied from 0 to 100% phase B within 15 min. The content of phase B was held for 7 min and then lowered to 0% within 20 s followed by equilibration of the column for 5 min. Quantitative determination of all compounds was performed by operating the mass spectrometer in ESI positive (T2 and HT2) and negative [DON and zearalenone (ZON)] ionization mode. Optimized instrument settings include capillary voltage 3.5 kV, source temperature 100°C; desolvation temperature 450°C; desolvation gas flow rate of 646 L hr^−1^ cone voltage, a cone gas flow rate of 72 L hr^−1^, and multiplier 650 V. Argon was used as the collision gas to increase the collision cell Pirani gauge to a reading of 8.74 e^−4^ mbar. Masslynx 4.0 software was used for data acquisition and processing. Quantification of the samples was carried out using a matrix of standards prepared in-house. The limit of detection for DON, ZON, T2, and HT2 was 10 μg kg^−1^.

### Statistical analysis

All data were analyzed using Genstat® Version 12.1 for Windows (VSN International Ltd, UK). The mist irrigated 2012 field experiment was analyzed separately from the 2010 and 2011 non-misted experiments. Where required, AUDPC, DNA, and mycotoxin data were square root or log10 transformed to normalize residuals. Analysis of variance was carried out on AUDPC, DNA, and mycotoxin data to determine significant differences and interactions between years at *P* < 0.05. Analysis of grain DNA from the 2011 and 2012 glasshouse experiments used a Kruscal–Wallis one-way ANOVA as the log10 transformation failed to normalize the residuals. The visualization of groupings between measured variables was explored using Principal Component Analysis (PCA) to create bi-plots using Microsoft® Excel 2010/XLSTAT-Pro (Version 2015.2.02.18135, Addinsoft, Inc., Brooklyn, NY, USA). Two separate PCA analyses were used to model the grouping of AUDPC with canopy and ear traits, DNA and mycotoxin levels and ear traits for the field and glasshouse experiments. Multiple Linear Regression (MLR) was used to model relationships between physiological traits and variation in AUDPC or mycotoxins. Data was grouped for experimental years to test regressions for position and parallelism. Multicolinearity between traits analyzed within the MLR was avoided and all variables were tested manually to ensure results were not confounded.

## Results

### Canopy and ear traits

Genotype and experiment interactions for canopy and ear traits were present in the field (Table 1) and glasshouse (Table 2). Trait means for individual genotypes from all experiments are shown as Supplementary Data (Supplementary Table 1) Number of tillers per plant, showed the largest variation within and between years, with a mean tiller number per plant of 5.8, 1.9, and 3.2 in the 2010, 2011, and 2012 field experiments. Longer and wider flag leaves were observed under misted field conditions with mean flag leaf length of 15.7, 16.6, and 17.3 cm and mean flag leaf width of 1.7 and 1.6 cm in 2010, 2011, and 2012 field experiments, respectively. Plants were taller in 2011 and 2012 with a mean height of 79.1 and 78.7 cm respectively, whilst in 2010 mean plant height was 70.8 cm. Number of spikelets was the ear trait that showed the highest variation between experiments with mean spikelets per ear between 19.3, and 22.5 in 2010 and 2011 and 2012. Mean ear length was found to be 9.5, 10.8, and 10.7 cm while spike density (spikelets cm^−1^) was consistently 2.1 in all field experiments (Table 1).

**Table 1:** Physiological traits of wheat genotypes, described by the mean, 95th percentile and maximum values, assessed in field experiments in 2010, 2011, and 2012.

	**Experiment**	**Mean**	**95th%**	**Max**	***P*****-value (SED;** ***df*****)**			**CV%**
					**Genotype**	**Experiment**	Genotype^*^Experiment	
**CANOPY**								
Plant height (cm)	2010	70.8	93.3	100.8	<0.001(2.40;58)	<0.001(0.87;58)	<0.001(3.35;58)	5.5
	2011	79.1	118.7	119.7				
	2012	78.7	106.3	111.4	<0.001(11.49;169)			17.9
Flag leaf length (cm)	2010	15.7	18.8	19.2	<0.001(0.80;58)	0.003(0.29;58)	0.002(1.13;58)	14.4
	2011	16.6	21.9	24.0				
	2012	17.3	23.3	25.0	<0.001(2.04;169)			11.6
Flag leaf width (cm)	2010	1.7	1.9	2.0	<0.001(0.05;58)	<0.001(0.02;58)	<0.001(0.07;58)	5.2
	2011	1.6	1.8	1.9				
	2012	1.6	1.9	2.2	<0.001(0.16;169)			11.8
No. of tillers per plant	2010	5.8	7.2	7.6	<0.001(0.32;58)	<0.001(0.11;58)	<0.001(0.45;58)	14.7
	2011	1.9	2.6	3.2				
	2012	3.2	5.2	6.8	<0.001(0.59;169)			22.6
**EAR**								
Ear length (cm)	2010	9.5	12.5	13.9	<0.001(0.33;58)	<0.001(0.12;58)	<0.001(0.46;58)	5.5
	2011	10.8	13.5	13.9				
	2012	10.7	12.4	13.2	<0.001(0.72;169)			8.3
Number of spikelets per ear	2010	19.3	21.4	22.2	<0.001(0.55;58)	<0.001(0.20;58)	<0.001(0.77;58)	4.5
	2011	22.5	25.8	26.6				
	2012	22.2	24.8	28.2	0.028(1.41;169)			7.8
Spike density (spikelets/cm)	2010	2.1	2.4	2.6	<0.001(0.05;58)	0.007(0.02;58)	<0.001(0.08;58)	4.4
	2011	2.1	2.5	2.7				
	2012	2.1	2.5	2.7	<0.001(0.12;169)			7.1

*SED, standard error of the difference; df, Degrees of freedom*.

**Table 2:** Area under the Disease Progress Curve (AUDPC), canopy and ear traits of wheat genotypes, described by the mean, 95th percentile and maximum values, assessed in glasshouse experiments in 2011 and 2012.

**Traits**	**Experiment**	**Mean**	**95th%**	**Max**	***P*****-value (SED;** ***df*****)**			**CV%**
					**Genotype**	**Experiment**	Genotype^*^Experiment	
AUDPC	2011	93.0	315.7	520.2	<0.001(71.0;78)	0.002(25.9;78)	0.033(95.9;78)	100.9
	2012	189.0	609.7	653.6				
**CANOPY**								
Plant height (cm)	2011	81.5	114.6	124.5	<0.001(3.47;78)	NS	0.010(4.90;78)	8.4
	2012	85.2	125.3	140.2				
Flag leaf length (cm)	2011	25.7	38.6	39.6	<0.001(2.31;78)	<0.001(0.88;78)	<0.001(3.26;78)	15.3
	2012	35.5	41.6	44.2				
Flag leaf width (cm)	2011	2.1	2.5	3.0	<0.001(0.11;78)	<0.001(0.04;78)	0.005(0.17;78)	9.8
	2012	2.6	3.2	3.8				
No. of tillers per plant	2011	9.1	14.2	16.0	<0.001(1.44;78)	<0.001(0.55;78)	<0.001(2.18;78)	28
	2012	11.6	23.0	29.0				
**EAR**								
Ear length (cm)	2011	15.5	20.6	21.9	<0.001(0.67;78)	0.006(0.26;78)	NS	8.4
	2012	16.5	21.4	27.5				
Number of spikelets per ear	2011	26.3	31.1	42.0	<0.001(1.20;78)	NS	<0.001(1.62;78)	8.8
	2012	26.2	32.2	34.0				
Spike density (spikelets/cm)	2011	1.7	2.0	2.1	<0.001(0.07;78)	0.009(0.02;78)	0.013(0.10;78)	8.4
	2012	1.6	2.0	2.3				
Awn length (cm)	2011	2.7	6.9	8.9	<0.001(0.46;78)	<0.001(0.17;78)	<0.007(0.70;78)	29.7
	2012	3.6	8.5	9.5				

*NS, not significant (P > 0.05); SED, standard error of the difference; df, Degrees of freedom*.

The numbers of tillers per plant were higher in the glasshouse than in the field experiments with a mean of 9.1 and 11.6 within 2011 and 2012 experiments, respectively. Leaves were also longer and wider in the glasshouse experiments than in the field with mean flag leaf length of 25.68 and 35.54 cm and mean width of 2.08 cm and 2.56 cm in 2011 and 2012, respectively. Awn length was only recorded in the glasshouse experiments and varied significantly within and between years (mean 2.7–3.6 cm; Table 2).

### Visual FHB symptoms and DNA of *Fusarium* and *Microdochium* spp.

FHB symptoms assessed as water-soaked lesions and bleaching of the glumes were present in each of the field experiments, with incidence of 100% of experimental plots. In the field trials under natural rainfall conditions in 2010 and 2011, AUDPC values of 46.5 and 8.8 were recorded respectively, which were lower than in 2012 when artificial misting was applied with mean AUDPC of 712 (Table 3). A significant year by genotype interaction (*P* < 0.001) for AUDPC was present for the field experiments in 2010 and 2011 with the same inoculation and significant differences (*P* < 0.001) were detected between genotypes for all years of experimentation.

**Table 3:** Area Under the Disease Progress Curve (AUDPC) and DNA of *Fusarium* and *Microdochium* spp. (pg ng^−1^ of total DNA) described by the mean, 95th percentile and maximum values in harvested grain of wheat genotypes in 2010, 2011, and 2012 field experiments.

						***P*****-value (SED;** ***df*****)**		
	**Experiment**	**Mean**	**95th%**	**Max**	**Genotype**	**Experiment**	Genotype^*^Experiment	**CV (%)**
AUDPC^a^	2010	6.8(46.5)	13.7(186.3)	17.9(320.8)	<0.001(0.87;58)	<0.001(0.32, 58)	<0.001(1.23;58)	30.9
	2011	2.9(8.8)	6.4(41.1)	7.9(61.9)				
	2012	24.2(712.0)	42.3(1792.9)	48.3(2336.8)	0.003(8.59;169)			42.6
*F.g^b^*	2010	−1.96(0.011)	−1.08(0.08)	−0.94(0.12)	NS	<0.001(0.18;58)	NS	31.3
	2011	−3.54(0.0003)	−1.50(0.03)	−1.27(0.05)				
	2012	−0.36(0.730)	0.47(2.98)	0.59(5.14)	<0.001(0.24;58)			82.7
*F.c^b^*	2010	−2.10(0.008)	−0.64(0.23)	−0.14(0.72)	<0.001(0.50;58)	0.001(0.18;58)	0.05(0.71;58)	30.9
	2011	−3.49(0.0003)	−1.22(0.06)	−0.80(0.16)				
	2012	−0.83(0.601)	0.30(2.07)	0.39(2.47)	<0.001(0.30;58)			45.1
*F.p^b^*	2010	−2.18(0.007)	−1.12(0.08)	−0.83(0.15)	0.025(0.45;58)	NS	0.008(0.63;58)	36.9
	2011	−2.00(0.010)	−1.18(0.07)	−1.03(0.09)				
*F.l^b^*	2010	−2.21(0.007)	−1.02(0.11)	−0.94(0.12)	<0.042(0.54;58)	<0.007(0.19;58)	NS	37.5
	2011	−2.76(0.002)	−1.52(0.03)	−1.24(0.06)				
*F.a^b^*	2010	−2.64(0.002)	−1.66(0.02)	−1.50(0.03)	NS	NS	NS	26.7
	2011	−2.65(0.002)	−2.11(0.01)	−2.05(0.01)				
*M.m^b^*	2010	−3.18(0.0007)	−1.71(0.02)	−1.47(0.03)	NS	<0.001(0.16;58)	NS	21.3
	2011	−3.92(0.00001)	−3.51(0.0003)	−2.28(0.01)				
	2012	−0.68(0.640)	0.32(2.09)	0.44(2.77)	<0.001(0.27;58)			49.5
*M.n^b^*	2010	−3.12(0.0008)	−1.60(0.03)	−1.37(0.04)	NS	<0.001(0.17;1)	NS	22.5
	2011	−3.84(0.00001)	−2.62(0.002)	−2.30(0.01)				
	2012	−0.84(0.420)	0.10(1.26)	0.35(2.21)	<0.001(0.26;58)			37.1

a *Square root transformation*,

b *Log10 transformation; Back transformed means in parentheses; NS, not significant (P > 0.05); SED, standard error of the difference; df, Degrees of freedom; Fg, Fusarium graminearum; Fc, F.culmorum; Fp, F. poae; Fl, F. langsethiae; Fa, F. avenaceum; Mm, M. majus; Mn, M. nivale*.

Significant interactions between genotypes and year of experimentation were detected for DNA of *F. culmorum, F. poae*, and *F*. *langsethiae* (Table 3). In 2010, the predominant species was *F. graminearum*, quantified at the highest concentration at 0.011 pg ng^−1^ of total DNA, followed by *F. culmorum, F. poae*, and *F. langsethiae* at 0.007 pg ng^−1^ of total DNA, whilst *F. avenaceaum* and *Microdochium* spp., were quantified at below 0.002 pg ng^−1^ of total DNA or lower in harvested grain samples. In contrast, in 2011, *F. poae* predominated with mean DNA concentration of 0.010 pg ng^−1^ of total DNA in harvested grain, followed by *F. avenaceum* while the rest of *Fusarium* and *Microdochium* spp. were found at 0.0003 pg ng^−1^ of total DNA or lower concentrations. *M. majus* or *M. nivale* were the least frequently detected pathogens during 2010 and 2011. In 2012, when the field experiment was artificially misted, DNA concentrations of *Fusarium* and *Microdochium* spp. quantified in grain samples were higher, with *F. graminearum* (0.73 pg ng^−1^ of total DNA) predominating, followed by *M. majus* (0.64 pg ng^−1^ of total DNA), *F. culmorum* (0.60 pg ng^−1^ of total DNA), and *M. nivale* (0.42 pg ng^−1^ of total DNA; Table 3).

Mean AUDPC by spray inoculation in the glasshouse in 2011 was 93.2 whilst in 2012 this was more than two-fold higher at 189 and a significant interaction was (*P* = 0.033) was present between experiment and genotype (Table 4). DNA concentrations were best described using Chi-square probability in terms of highest-ranking species between and within years of experimentation. In 2011, *F. langesthiae* predominated, followed by *F. graminearum*, while *F. avenaceum* had ranked the lowest DNA concentration in harvested grain. In contrast, in 2012, *F. avenaceum* followed by *F. culmorum* ranked highest DNA concentrations while *F. langesthiae* ranked the lowest DNA concentration. *M. majus* or *M. nivale* DNA were detected but not found in quantifiable amounts in the glasshouse experiments.

**Table 4:** DNA of *Fusarium* spp. and *Microdochium* spp., described by the mean rank and Chi-square probability, of wheat genotypes assessed in glasshouse experiments in 2011 and 2012.

	**Experiment**	**Mean rank**	**Chi-square probability**
*F. graminearum*	2011	59.71	0.203
	2012	51.94	
*F. culmorum*	2011	40.33	<0.001
	2012	73.15	
*F.poae*	2011	54.48	0.438
	2012	57.66	
*F. langsethiae*	2011	68.16	<0.001
	2012	42.70	
*F. avanaceum*	2011	30.50	<0.001
	2012	83.38	
*M. majus*	2011	nq	nq
	2012	nq	
*M. nivale*	2011	nq	nq
	2012	nq	

*nq, Not quantifiable*.

### Mycotoxin concentration

Analysis of variance revealed significant interactions between genotype and experiment for DON and HT2 +T2 contamination in harvested grain in 2010 and 2011 field experiments (Table 5). The predominant mycotoxin in both misted and non-misted experiments was DON with mean concentration of 234.42, 19.40, and 619.06 μg kg^−1^ during 2010, 2011, and 2012 field experiments, respectively. ZON was found at the highest concentration in 2012 (mean of 203.23 μg kg^−1^) when misting was applied. Concentration of HT2+T2 in 2010 was more than four-fold higher than in 2011 with a mean of 48.98 μg kg^−1^ (Table 5).

**Table 5:** Deoxynivalanol (DON), HT2 + T2, and zearalenone (ZON) concentrations (μg kg^−1^) described by the mean, 95th percentile, and maximum values in harvested grain of wheat genotypes in field experiments in 2010, 2011, and 2012.

	**Experiment**	**Mean**	**95th%**	**Max**	***P*****-value (SED;** ***df*****)**			**CV (%)**
					**Genotype**	**Experiment**	Genotype^*^Experiment	
DON^a^	2010	2.37(234.42)	3.04(1083.48)	3.13(1352.23)	<0.001(0.22;58)	<0.001(0.08;58)	NS	20.7
	2011	1.29(19.40)	2.38(239.88)	2.72(535.79)				
	2012^b^	2.78(619.06)	3.14(931.25)	3.14(1194.00)				
ZON^a^	2010	1.08(15.14)	1.52(33.11)	1.71(51.56)	NS	0.004(0.03;58)	NS	12.2
	2011	1.00(10.47)	1.13(13.49)	1.42(23.80)				
	2012^b^	2.31(203.23)	2.91(811.18)	2.93(855.00)				
HT2+T2^a^	2010	1.69(48.98)	2.23(169.15)	2.37(234.88)	<0.001(0.13;58)	<0.001(0.05;58)	0.006 (0.19; 58)	17.0
	2011	1.05(11.22)	1.35(22.39)	1.85(71.06)				

a *Log10 transformation. Back transformed means shown in parentheses, NS, not significant (P > 0.05); SED, standard error of the difference; df, Degrees of freedom*.

b *Mycotoxin contamination in 2012 field experiment quantified using flour combined together from three replicates of 30 selected lines*.

### Principal component analysis (PCA)

**Figure 1:**
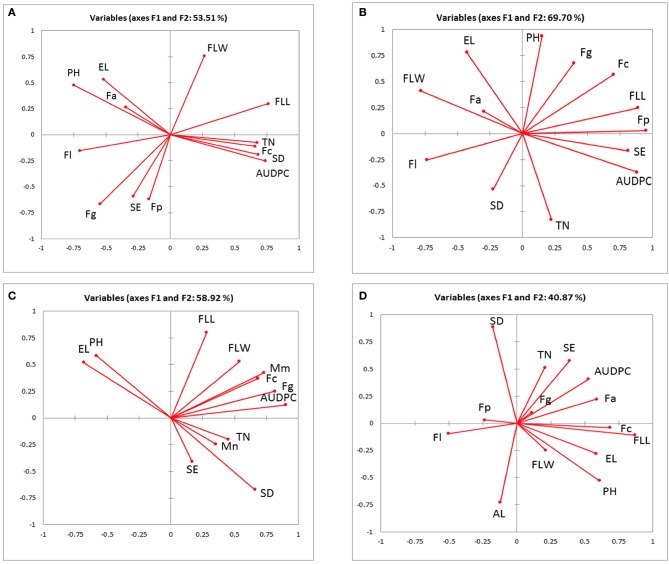
Biplot of the principal component analysis of the Area under the disease progress curve (AUDPC), pathogen DNA (pg ng^−1^), measured canopy and ear traits from **(A)** 2010 **(B)** 2011 **(C)** 2012 field experiment **(D)** 2011–2012 glasshouse experiments. EL, ear length; FLL, flag leaf length; FLW, flag leaf width; PH, plant height; SE, spikelets per ear; SD, spikelet density; TN, tiller number; AL, awns length; Fg, *Fusarium graminearum*; Fc, *F. culmorum*; Fp, *F. poae;* Fa, *F. avenaceum*; F1, *F. langsethiae*; Mm, *Microdochium majus*; Mn, *M. nivale*.

The associations between AUDPC, pathogen DNA, canopy and ear traits in 2010, 2011, 2012 field experiments and 2011 and 2012 glasshouse experiments were visualized using principal component analysis (PCA) accounting for 53.5, 69.7, 58.9, and 40.9% of the total variance, respectively (Figures 1A–D). AUDPC in 2010 grouped closely with tiller numbers, flag leaf length, spikelet density and *F. culmorum* and was negatively associated with ear length, plant height and *F. avenaceum* (Figure 1A). In 2011, AUDPC clustered with flag leaf length, spikelets per ear and *F. poae* (Figure 1B). In 2012, AUDPC was associated with DNA of *M. majus, F. graminearum*, and *F. culmorum* (Figure 1C). Most consistently, there were negative associations between *F. poae* and flag leaf width and *F. langsethiae* and flag leaf length (Figures 1A–D) and positive associations between ear length and plant height. Tiller numbers, *M. nivale* DNA, spikelet density and spikelets per ear grouped closely in 2012. *F. culmorum* DNA and flag leaf length separated in a distinct group in the PCA analysis of the glasshouse experiment (Figure 1D).

**Figure 2:**
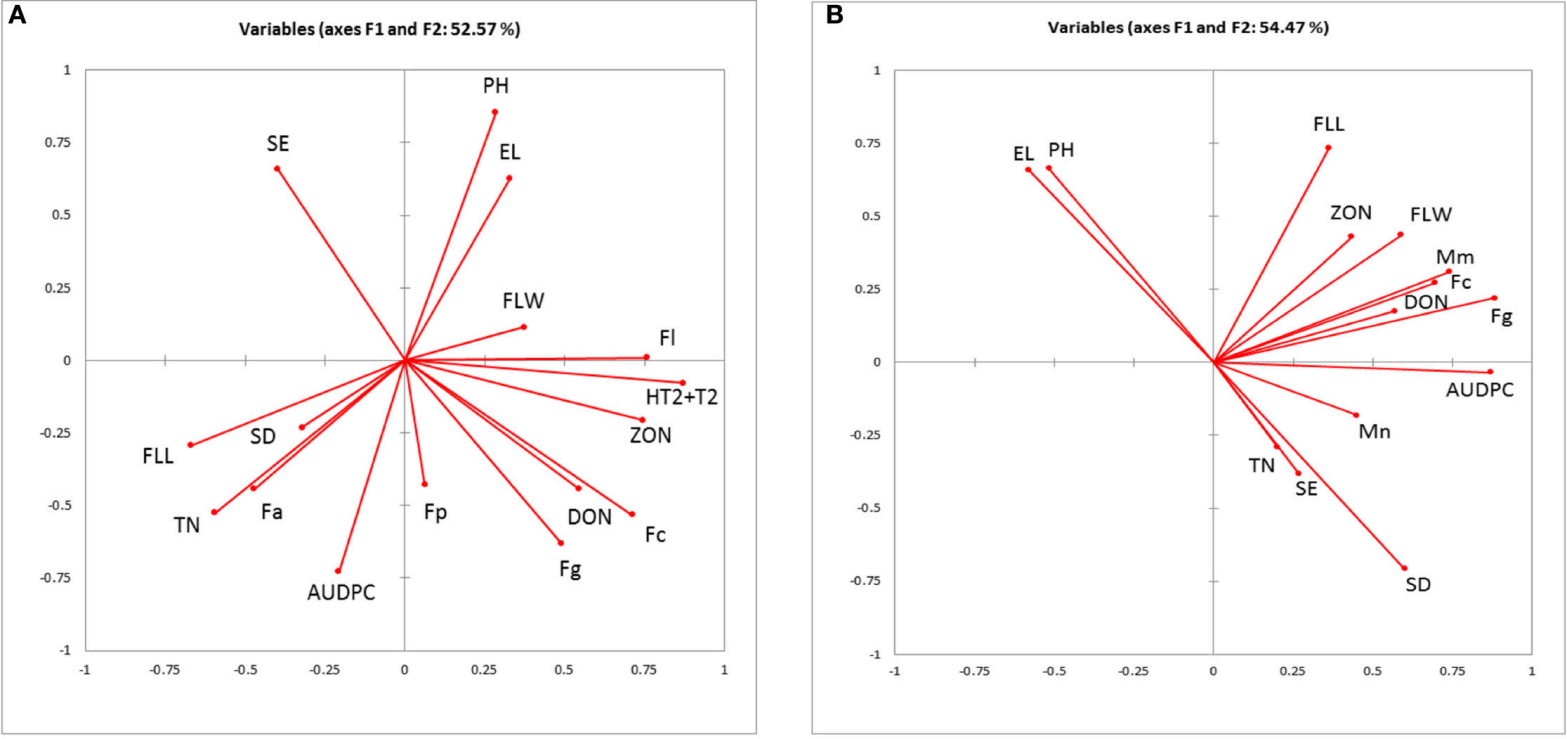
Biplot of the principal component analysis of the Area under the disease progress curve (AUDPC), measured ear traits, quantified fungal DNA and mycotoxins from **(A)** 2010 and 2011 field experiments; **(B)** 2012 field experiment. El, ear length; SE, spikelets per ear; SD, spikelet density; Fa, *F. avenaceum*; Fc, *F. culmorum*; Fg, *F. graminearum*; Fl, *F. langsethiae*; Fp, *F. poae*; Mm, *M. majus*, Mn, *M. nivale*; DON, deoxynivalenol, HT2+T2 and ZON, zearalenone.

PCA for canopy and ear traits, AUDPC, DNA, and mycotoxin concentration in 2010–2011 and 2012 field experiments showed 52.46 and 54.47% of collective variance, respectively (Figures 2A,B). Plant height and ear length clustered together in both PCA models. In both models, DNA of *F. graminearum* and *F. culmorum* clustered with DON although in 2012 *M. majus* was also associated with *F. culmorum*. ZON grouped more closely with *F. langesthiae*, HT2-T2 and flag leaf width in 2010–2011 and flag leaf length and width in 2012.

### Predictions of AUDPC and mycotoxins using canopy and ear traits and pathogen DNA

MLR was performed to predict AUDPC using canopy and ear traits and pathogen DNA identified from the PCA analysis. Consistently flag leaf length, number of tillers per plant and spikelet density associated positively in prediction of AUDPC in addition to DNA of *F. culmorum* in 2010 (*R*^2^ = 0.61, *P* < 0.001) and DNA of *F. graminearum* in 2012 (*R*^2^ = 0.78, *P* < 0.001). In 2011, when *F. poae* was related to AUDPC, canopy traits were not significant and only number of spikelets per ear negatively influenced the disease accounting for 29% of the variance (*P* < 0.001). The analysis of data from the glasshouse experiments showed that flag leaf length, number of tillers per plant, number of spikelets per ear and DNA of *F. avenaceum* accounted positively toward variation in AUDPC for both years with the data fitting a common line (*R*^2^ = 0.44, *P* < 0.001; Table 6).

**Table 6:** Multiple linear regression of physiological traits and *Fusarium* spp. DNA (pg ng^−1^ of Total DNA) in grain on Area Under the Disease Progress Curve (AUDPC) of wheat genotypes in field experiments 2010, 2011, & 2012 and 2011 & 2012 glasshouse experiments.

**Model**	**Equation**	***R*^2^**	***P*-value**
**AUDPC**			
**Field**			
2010	y = 48.3 (log10 Fc) + 19.92 (TN) + 13.67 (FLL) + 70 (SD) −351.5	0.61	<0.001
2011	y = 16.22 (log10 Fp) – 2.28 (SE) + 90.7	0.29	<0.001
2012	y = 125.9 (log10 Fc) + 64 (log10 Fg) + 98.3 (TN) + 64 (FLL) + 743 (SD) −2442	0.78	<0.001
**Glasshouse**			
2011& 2012	y = 15.33 (log10 Fa) + 7.05 (TN) + 6.9 (FLL) + 11.71 (SE) −459	0.44	<0.001

*TN, Tiller number; FLL, flag leaf length; SD, spikelet density; SE, spikelets per ear; Fc, F. culmorum DNA; Fp, F. poae DNA; Fa, F. avenaceum DNA*.

MLR analysis of mycotoxin concentrations revealed that the data fitted best parallel lines for 2010 and 2011 with the same slope but different intercepts. The variation in DON (*R*^2^ = 0.43, *P* < 0.001) was explained best by DNA of *F. graminearum* and *F. culmorum*. In 2012 when the field was misted, variation in DON concentration (*R*^2^ = 0.48, *P* < 0.001) was accounted for by DNA of *F. graminearum* and number of spikelets per ear. ZON concentration in harvested grain was positively related to DNA of *F. culmorum* in 2010 and 2011 experiments and HT2+T2 contamination with the data fitting a common line for both years (*R*^2^ = 0.48, *P* < 0.001). In 2012, variation in ZON concentration (*R*^2^ = 0.54, *P* < 0.001) was predicted by DNA of *F. graminearum* and *F. culmorum*. HT2+T2, quantified in 2010 and 2011 field experiments were associated with DNA of *F. langsethiae* and were negatively related to the number of spikelets per ear accounting for 53% of the variance with the data fitting best parallel lines with different intercepts for each year (*P* < 0.001; Table 7).

**Table 7:** Multiple Linear Regression of physiological traits and *Fusarium* spp. DNA (pg ng^−1^ of Total DNA) on mycotoxin concentration in harvested grain in field experiments 2010, 2011, and 2012.

**Model**	**Experiment**	**Equation**	***R*^2^**	***P*-value**
Log10 DON	2010	y = 0.67 (log10 Fg) + 0.23(log10 Fc) + 4.24	0.43	<0.001
	2011	y = 0.67 (log10 Fg) + 0.23(log10 Fc) + 3.39		
	2012	y = 0.46 (log10 Fg) + 0.09(SE) + 0.84	0.48	<0.001
Log10 ZON	2010 & 2011	y = 0.56 (log10 Fc) + 0.29(log HT2+T2) + 1.20	0.48	<0.001
	2012	y = 0.28 (log10 Fg) + 0.24(log10 Fc) + 2.18	0.54	<0.001
Log10 HT2+T2	2010	y = 0.67 (log10 Fl) – 0.05 (SE) + 3.90	0.53	<0.001
	2011	y = 0.67 (log10 Fl) – 0.05 (SE) + 3.08		

*SE, Spikelets per ear; Fg, F. graminearum DNA; Fl, F. langsethiae DNA; Fc, F.culmorum DNA*.

## Discussion

Passive resistance mechanisms act through the expression of morphological traits to allow the host to escape contact with the pathogen or avoid successful colonization once contact has been established. From an ecological perspective, canopy and ear traits may also alter pathogen co-occurrence and niche occupation with consequences for disease severity or mycotoxin accumulation. In each field experiment described here, distinct canopy and ear traits interacting with the environment contributed to the progress of FHB, expressed as AUDPC, by influencing the dispersal, spread, and infection by *Fusarium* pathogens and subsequently toxin accumulation within grains. Ground-originating infectious ascospores or conidia produced by FHB pathogens are effectively dispersed to ears by wind and rain splash and can be spread further by insect vectors (Manstretta et al., 2015; Drakulic et al., 2017). In this work, greater tiller numbers, longer flag leaves, and higher spikelet density were consistently correlated with AUDPC by *F. culmorum* and *F. graminearium*. The first two traits are typical of dense canopies and in relation to disease epidemics may favor vertical spore movement on leaf layers when aided by rain splash. Indeed, under field conditions, spores of *F. culmorum* have been shown to be splash dispersed vertically toward the ear during rainfall events (Jenkinson and Parry, 1994; Hörberg, 2002). Furthermore, longer flag leaves are likely to result in a greater and more frequent contact between leaf surfaces and the ears of neighboring plants thus facilitating horizontal dispersal whilst dense spikelets are likely to provide a microclimate of increased humidity favoring fungal growth, spread, and sporulation within individual heads. Mesterhazy (1995) was one of the first researchers to note spikelet density as a potential passive resistance trait and here we confirm his observations and provide evidence of the importance of this trait for disease progression. The quantity, quality, and timing of rainfall during anthesis in the field is most likely to alter the dispersal and spread of available spores to ears at anthesis. *Microdochium* species contributed to FHB disease only in 2012 indicating that misting at anthesis played a major role for the successful dispersal and infection by these species. Under misting conditions, *M. majus* was able to co-exist with *F. culmorum* and grouped closely with *F. graminearum* and AUDPC in the PCA. In contrast, *M. nivale* clustered with tiller numbers and spikelet density although fungal biomass of this species was not significantly associated with FHB disease development. Previous studies have reported similar associations between *F. graminearum* and *M. majus* in field conditions (Xu et al., 2005; Audenaert et al., 2009), however other studies (Xu et al., 2008) attributed the positive association among species to similar responses to climatic conditions rather than to direct interaction.

In 2011 when AUDPC was associated with *F. poae*, canopy traits had no influence on FHB progress but instead ear traits, specifically number of spikelets per ear, were negatively related to FHB development and severity. The effect of spikelets per ear on FHB caused by different pathogens or indeed on their mycotoxins is at best described as unclear. For example, Liu et al. (2007) reported lack of significant relationship between spikelets per ear and AUDPC whilst here we show that the relationship is negative for FHB by *F. poae* and HT2+T2 produced by *F. langsethiae*, but positive for *F. avenaceum* and DON by *F. graminearium* under misted conditions in 2012. It seems that the relationships between ear traits and FHB disease and mycotoxins are depending on the range of dispersal mechanisms of the pathogens, their interaction with other species and the environment. For example, in addition to asexual conidia, *F. graminearum* is capable of producing ascospores that rely on wind or air dispersal once ejected from the perithecium on ground debris and therefore canopies may play less significant role in the spatial aspect of the host-pathogen interaction. Other species such as *F. poae, F. avenaceum*, and *F. langsethiae* have been previously shown able to be transmitted by insects (Drakulic et al., 2017) whilst both *F. langsethiae* and *F. graminearium* can spread more rapidly within heads and produce more toxins under insect infestation (Drakulic et al., 2015, 2016). Thus it is likely that for the *Fusarium* species with diverse mechanisms of transmission and spread, ear traits may play equally or more important role in disease and toxin production outcome.

Here we report for the first time significant relationships between flag leaf length, tiller numbers, and AUDPC of FHB consistently in field and glasshouse experiments, using different methods of inoculation thus we speculate that these two traits apart from influencing epidemiological phases of the disease may be contributing to FHB susceptibility or compromised Type I resistance to initial infection by *Fusarium graminearum, F. culmorum*, and *F. avenaceum*. Whilst we propose this hypothesis based on repeated field and glasshouse experimentation we also consider that limited number of genotypes were used in our studies in order to allow for the extensive, phenotypic analysis of a range of traits to be performed. Thus to fully de-convolute the identified passive traits from genetic resistance or identify genetic linkages further genetic association studies using a larger DH wheat population will be required. Recently, fine mapping of a major QTL for increased flag leaf width on chromosome 5A showed a tight linkage to *Fhb5* for Type I resistance to FHB in wheat (Xue et al., 2013). No known reports of genetic linkage between FHB and tiller inhibition (*Tin*) genes have yet been published, however, semi dwarf wheat cultivars have been shown to produce more fertile tillers and higher grain yield than taller wheat varieties (Daoura et al., 2014). Mahboob et al. (2002) also reported that genotypes with *Rht1* produced significantly greater number of tillers per plant thus contributing to higher yield per plant. Plant height alleles segregating within the DH population used here include *Rht-B1b* and *Rht-D1b* (formerly termed *Rht1* and *Rht2*) (Foulkes, Pers Comms) which have been previously linked to FHB susceptibility (Srinivasachary et al., 2009), therefore, the positive influence of tiller numbers on disease found in the present studies may also be indirectly related to the presence of the *Rht* alleles within the tested genotypes making these genotypes more susceptible.

In agreement to other studies (Dornbusch et al., 2011; Yang et al., 2016) we observed significant interactions between genotypes and different environments for canopy traits and in particular flag leaf characteristics indicating high phenotypic plasticity as shown by the wide variation in the expression of these traits in different seasons. Numerous studies by Mei et al. (2005), Ding et al. (2011), and Wang et al. (2012) have suggested pleiotropic effects and linked loci simultaneously affecting flag leaf morphological traits and yield potential in cereals. Furthermore, Fan et al. (2015) reported several quantitative trait loci (QTL) on chromosome 4B, 6B, and 5B simultaneously controlling flag leaf related traits (flag leaf length, width, and area) and yield related traits in wheat. Thus as the flag leaf contributes significantly to photosynthesis during grain filling and final yield, reducing its length to enable disease escape or passive resistance such as avoidance is likely to result in a significant trade-off with yield. Similarly, tiller production and particularly fertile tiller survival have been shown to determine final ear and grain numbers thus play a key role in final grain yield formation in wheat (Sharma, 1995; Destro et al., 2001; Xie et al., 2015). Potential trade-off between passive resistance and yield traits are therefore likely, thus uncoupling any underlying genetic linkages can improve breeding new varieties without the costs of increased susceptibility.

Overall mycotoxin accumulation was significantly related to DNA of the main producers and influenced by environmental conditions altered by the addition of misting to natural rainfall in the last season of our work. DON in harvested grain was related significantly to fungal biomass of the two producers, *F. graminearum* and *F. culmorum* rather than canopy or ear traits. Tiller number has been previously reported to have a negative relationship with DON contamination of grain as tillers were shown to accumulate less mycotoxins than main stem ears when a conidia suspension was applied (Gautam et al., 2012) with the authors suggesting that this was due to main stem ears reaching anthesis earlier than tillers, allowing the pathogen a longer period to colonize wheat heads and produce DON. The study concluded that increased tiller numbers would reduce DON contamination in grain (Gautam et al., 2012) which we are unable to confirm with the findings from our work. Instead, we would suggest that greater tiller numbers contributing to AUDPC by *F. culmorum* or *F. graminearum* are likely to result in increased DON via accumulation of fungal biomass in grain. Under misting in 2012, ZON was produced by *F. culmorum* and *F. graminearium* and contamination was ten-fold higher than under natural rainfall. In contrast in 2010 and 2011, ZON was associated with *F. culmorum* and HT+T2 grouping together.

It was expected that ear traits such as awn length may influence disease or mycotoxin accumulation but the results from our studies in the glasshouse experiments, with limited number of genotypes, agree with reports that have found no relationship of awn presence or length with FHB (Buerstmayr et al., 2000; Somers et al., 2003; Liu et al., 2007). Ear length and spikelets per ear were poorly correlated with each other and although negative correlation between ear length and FHB severity has been previously found (Suzuki et al., 2012) our results indicate that spikelet density (number of spikelets per cm of ear) may be more appropriate trait to correlate with AUDPC and fungal biomass in future studies.

Our results suggest that varieties with lower tiller numbers, shorter flag leaves and less dense ears may be able to avoid FHB disease caused by *Fusarium* and *Microdochium* species aided by the spatial architecture of their canopies and ears. However, decisions to alter or manipulate these traits to avoid FHB should consider the potential trade-off with biomass and grain production traits resulting in lower yields.

In conclusion, important canopy and ear traits were identified in these studies associated with increased FHB disease progression. Canopy traits able to influence the vertical dispersal of inoculum from crop debris such as tiller number and the horizontal spread between plants such as flag leaf length were associated positively with AUDPC of FHB. Similarly, ear traits creating more conducive environment for infection and colonization such as spikelet density favored disease development when caused by *F. graminearum* and *F. culmorum*. In contrast to disease, fungal biomass of the producers and number of spikelets per ear were associated with mycotoxin accumulation by *F. graminearum, F. culmorum* and *F. langsethiae*. Since tiller numbers and flag leaf length were found to correlate with the disease using ground and head inoculations, future studies to confirm genetic relationships with FHB susceptibility or resistance will be needed. Agronomy practices or cultivars with shorter flag leaves, lower tiller numbers together with less dense ears may escape disease in individual seasons caused by *F. graminearum, F. culmorum, F. avenaceum*, and *Microdochium* spp. although a trade off with yield by selecting or manipulating agronomy for these traits is likely and therefore the focus must remain on the improvement of genetic resistance to FHB.

## Author contributions

SJ, AF, JF, and RR conceived the project and its components. SJ and AF performed the experiments and collected the data. SJ, RR, and RL developed and optimized the LCMS/MS assay. SJ, AF, and RR performed the data analysis and wrote the manuscript. JF, RL, and DS commented on the manuscript.

### Conflict of interest statement

The authors declare that the research was conducted in the absence of any commercial or financial relationships that could be construed as a potential conflict of interest.

## Supplementary material

Data Sheet 1 Field experimental design.

Supplementary Table 1 Trait means for genotypes from individual experiments.
